# Synthesis of Controlled-Release Calcium Peroxide Nanoparticles Coated with Dextran for Removal of Doxycycline from Aqueous System

**DOI:** 10.3390/polym14183866

**Published:** 2022-09-15

**Authors:** Nurul Nazihah Amerhaider Nuar, Siti Nurul Ain Md. Jamil, Fan Li, Intan Diana Mat Azmi, Pen-Chi Chiang, Thomas Shean Yaw Choong

**Affiliations:** 1 Department of Chemistry, Faculty of Science, Universiti Putra Malaysia (UPM), Serdang 43400, Selangor, Malaysia; 2 Centre of Foundation Studies for Agricultural Science, Universiti Putra Malaysia (UPM), Serdang 43400, Selangor, Malaysia; 3 Center of Sustainable Research, Department of Chemical and Environmental Engineering, Faculty of Engineering, Universiti Putra Malaysia (UPM), Serdang 43400, Selangor, Malaysia; 4 Graduate Institute of Environmental Engineering, National Taiwan University, Taipei City 10673, Taiwan; 5 Institute of Tropical Forest and Forest Products (INTROP), Universiti Putra Malaysia (UPM), Serdang 43400, Selangor, Malaysia

**Keywords:** controlled release, calcium peroxide, nanoparticles, dextran, doxycycline, organic polymer coating, fenton reaction

## Abstract

Nanoscale calcium peroxide (nCP) has turned out to be one of the effective and environmentally friendly approaches for wastewater remediation purposes. The rapid hydrolysis of nCPs and burst oxygen release caused by the high surface-to-volume ratio of nCPs could surpass the appropriate demand for oxygenation and pollutant degradation in the aqueous system. Thus, coated oxidants (COs) have been prepared using polymeric materials to ensure long-term efficacy and slow-release capability. Therefore, the nCPs were first prepared using dextran as a stabilizer to prevent irreversible agglomeration by the chemical precipitation method and had an average mean size of 2.33 ± 0.81 nm. The synthesized nCPs were then coated with dextran to produce dextran-coated nCPs. Their characteristics and effectiveness in doxycycline (DOX) degradation were assessed. The characterization of nCPs and dextran-coated nCPs was performed using X-ray diffractometry (XRD), field emission scanning electron microscopy (FESEM), fourier transform infrared spectroscopy (FTIR), Brunauer, Emmett and Teller analysis (BET), dynamic light scattering (DLS) and thermogravimetric analysis (TGA) techniques. This work suggests that dextran-coated nCPs are beneficial in wastewater treatment practice in terms of the long-term efficacy of DOX degradation potential.

## 1. Introduction

Antibiotics produce pollutants that endanger human health and the environment due to their massive productions and applications [1]. Doxycycline (DOX), which is grouped under tetracycline antibiotics, has been used in combination with other drugs to minimize COVID-19 inflammation in 2020 [2]. Thus, the demand and production of DOX have increased significantly. In the class of antibiotics, DOX is also recognized as highly refractory and persistent in aquatic systems [3]. As a result of high consumption, there is a possibility that DOX enters the sewage treatment plant via the urine of COVID-19 patients. In addition, the occurrence of DOX residues in wastewater is considerably exacerbated by the pharmaceutical industry [4].

Several approaches have been proposed for the removal of DOX antibiotics from aqueous solutions such as electrocoagulation [5], adsorption using rice husk [6] and adsorption by manganese oxide (MnO_x_) nanoparticle-loaded biochar (BC) [7]. Advanced oxidation processes (AOPs) break down antibiotics or transform them into compounds with a low molecular weight. This process can potentially reduce the antibiotics′ inhibitive action on bacteria, as well as increase their biodegradability and rate of elimination [8]. Fenton oxidation techniques are among the AOPs that are frequently utilized in wastewater treatment. The wastewater is treated with Fenton reagents (Fe^2+^ and H_2_O_2_), which can react to create hydroxyl radicals (•OH) in the Equation (1) [9]. The advantages of these procedures include the reagents′ inherent safety and environmental friendliness, generally straightforward mode of operation, quick reaction times and the lack of any mass transfer limitations. Hydroxyl radicals are used to oxidize refractory substances, producing the harmless by-products H_2_O and CO_2_ [10].

Fe(II) + H_2_O_2_ → •OH + OH^−^ + Fe(III) (1)

In comparison to dissolved hydrogen peroxide (H_2_O_2_), calcium peroxide (CaO_2_) is a solid and stable peroxide that is ideal for environmental remediation [11]. Equations (2) and (3) describe CaO_2_ as an oxygen-releasing substance that gradually releases oxygen species such as oxygen and H_2_O_2_ when dissolved in water [12]. Thus, CaO_2_ is a promising solid peroxide. It shows significant degradation of various contaminants. For instance, CaO_2_ has been applied to destruct the endocrine-disrupting compound [13], sulfanilamide [14], non-steroidal anti-inflammatory drugs (NSAIDs) [15] and antibiotics [16,17].

CaO_2_ + H_2_O → ½ O_2_ + Ca(OH)_2_ (2)

CaO_2_ + 2H_2_O → H_2_O_2_ + Ca(OH)_2_ (3)

In recent years, nanoscale CaO_2_ (nCP) has proven to be one of the most effective and environmentally friendly approaches to wastewater remediation. nCPs have better dispersion and transport capacity than commercial CaO_2_ [18]. During nanoparticle synthesis, an increase in the surface area to volume ratio of CaO_2_ particles results in an increase in the capacity to produce oxygen in a given amount of time. According to Khodaveisi et al. (2011), nCPs oxidized silver nanoparticles more quickly than microscopic CaO_2_ [19]. This is explained by the fact that nCPs have a higher surface area to volume ratio, resulting in a fast rate of reaction and rapid degradation, resulting in a higher H_2_O_2_ concentration for the oxidation of silver nanoparticles. However, in real-world applications, the rapid hydrolysis of nCPs and the abrupt release of oxygen from these particles results in hyperoxic conditions and an alkaline pH [20]. Therefore, polymeric materials are often used to produce coated oxidants that can ensure long-term effectiveness and provide slow-release function. Coating polymers should be biodegradable, well dispersed, biosoluble, biocompatible, non-toxic and unaffected by nanoparticles, so they can be utilized in a living system.

Polymers-based nanoparticles have recently received extensive attention, especially in drug delivery applications. For example, Javad et al. (2021) have studied chitosan-based nanomaterials as drug delivery for cancer treatments [21]. The studies have revealed that with the application of chitosan, the bioavailability, drug loading efficiency, releasing capacity and encapsulation efficiency of the nanomaterials are significantly enhanced. Next, Jimin Lee and Hongkee Sah (2022) synthesized spongelike poly-d,l-lactide-co-glycolide (PLGA) nanoparticles and successfully increased the drug encapsulation efficiency [22]. Their studies showed that with the application of PLGA, the nanoparticles have aggregative stability as the size of distribution data remains unchanged after a week of storage. Kuskov et al. (2016) studied the toxicity of polyvinyl pyrrolidone (PVP)-based nanoparticles for drug delivery systems [23]. They concluded that PVP is not toxic and has high biocompatibility for drug delivery systems, and with the application of PVP, nano-aggregates with high stability can be synthesized. Furthermore, polymers have also been applied as surface stabilizers to prevent irreversible agglomeration during synthesization and create stable dispersion such as polyethylene glycol (PEG) [23,24], polyvinyl pyrrolidone (PVP) [24,25,26,27], polyvinyl alcohol (PVA) [20], dextran [28,29,30], chitosan [31,32] and diethylene glycol monomethyl ether [24]. Among them, dextran (C_6_H_10_O_5_)_n_ has been used in several studies as surface stabilizers of nanoparticles. Dextran is a complex, branched polysaccharide-polymer chain unit that can range in length from around 1000 to 2,000,000,000 Da. It is mostly composed of linear chains of a-1,6-linked gluco-pyranose residues. Furthermore, it is one of the natural polysaccharides that has great solubility in water and is non-toxic and biocompatible [33]. In order to improve the efficiency of various metal oxides in the treatment of wastewater, dextran has been utilized. This is possible due to the biocompatibility features that it offers. Other nanoparticles have been effectively encapsulated by dextran for a wide range of applications, such as controlled release and drug delivery.

In this study, the nCPs were prepared using dextran (DEX) as a surface stabilizer and designated as nCPs (2g DEX). The nCPs (2g DEX) were further used for the synthesis of controlled-release calcium peroxide nanoparticles known as dextran-coated nCPs (2g DEX) by coating them with dextran. Several sample characterizations were carried out to assess the impact of polymer coating on the synthesized dextran-coated nCPs (2g DEX). The H_2_O_2_ release patterns of the synthesized dextran-coated nCPs (2g DEX) were outlined to categorize the controlled-release efficiency of the synthesized nCPs and dextran-coated nCPs (2g DEX) under identical conditions. Subsequently, the characterizations of the nCPs(2g DEX) and the dextran-coated nCPs (2g DEX) were thoroughly evaluated based on the differences in the H_2_O_2_ release pattern. Lastly, the efficiency of the prepared dextran-coated nCPs (2g DEX) was studied by the performance of DOX degradation in a simulation of DOX-contaminated water remediation.

## 2. Materials and Methods

### 2.1. Materials

Calcium peroxide (CaO_2_, 65%), dextran (C_6_H_10_O_5_)_n_ and doxycycline hydrochloride (C_22_H_25_ClN_2_O_8_) were purchased from Alfa Aesar, Ward Hill, MA, USA. Calcium chloride (CaCl_2_, ≥99.5%), hydrogen peroxide (H_2_O_2_, 30%), ammonia solution (NH_4_OH, 25 wt%), sodium hydroxide (NaOH), ethyl alcohol (C_2_H_5_OH, 95%) and hydrochloric acid (HCl), 37%) were acquired from R&M Chemicals Sdn. Bhd. (Semenyih, Malaysia). Iron (II) sulfate (FeSO_4_.7H_2_O, ≥98%) was purchased from BDH Chemicals Ltd., Poole, UK, and sodium sulphite (Na_2_SO_3_, ≥98%) was procured from Sigma Aldrich (St Louis, MO, USA). Cerium (IV) sulfate tetrahydrate (CeO_8_S_2_.4H_2_O, ≥98%) was purchased from Acros Organics, Geel, Belgium. Analytical reagent grade chemicals were used in this project, all of which were used as received. Distilled water was used throughout the experiments to prepare the solutions. The pH of the solution used for the synthesis of the nCPs was adjusted with 1M NaOH solution and 1M HCl solution.

### 2.2. Synthesis of nCPs with Dextran

**Figure 1:**
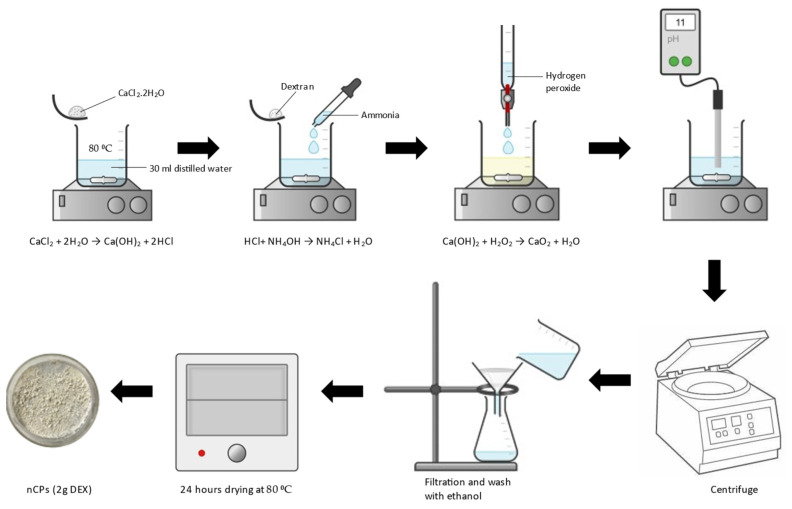
Schematic diagram on synthesis of nCPs with dextran.

The synthesis of nCPs was carried out using a chemical precipitation approach as described in previous studies with a few modifications [34]. An amount of 0.01 mol of CaCl_2_ was added into the solution containing 30 mL distilled water and heated until 80 °C. Then, 2 g of dextran was added to the solution, followed by 7 mL of ammonia solution. A total of 14 mL of H_2_O_2_ was added dropwise under constant stirring at 300 rpm after a reaction time of 3 min. The mixture was stirred for 2 h at constant stirring until a yellow beige solution was obtained. A gradual amount of 1M sodium hydroxide was added into the solution drop by drop, slowly touching the vessel′s walls and the pH was adjusted to 11–12. The mixture turned into a white suspension after the NaOH was added. The white precipitate was separated by centrifugation, and the powder was rinsed with ethanol three times. The resulting precipitate was dried in an evacuated oven for 24 h at 80 °C. The obtained nanocomposite was labelled as nCPs (2g DEX). A schematic diagram on the synthesis method is shown in Figure 1.

### 2.3. Synthesis of nCPs Coated with Dextran

**Figure 2:**
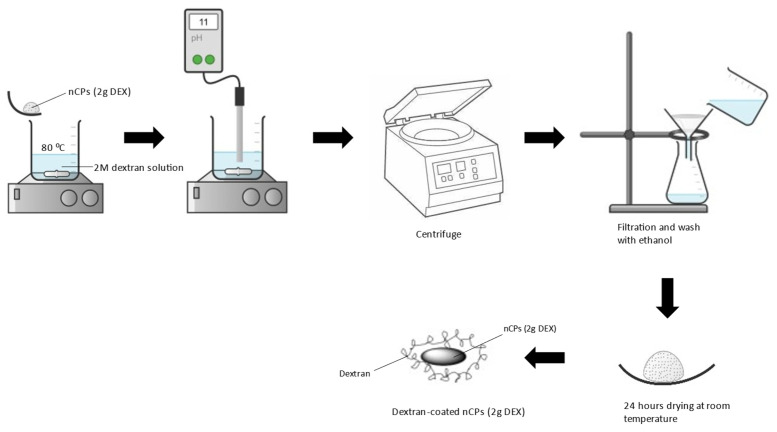
Schematic diagram on synthesis of nCPs coated with dextran.

Dextran-coated nCPs were prepared by adding nCPs (2g DEX) in 2M dextran solution and the solution was heated up to 90 °C. The solution was stirred at 300 rpm/min for 1 h. Then, 1M NaOH was added dropwise until a solution with a pH of 11–12 was obtained. The suspension was centrifuged and the precipitates were rinsed three times with ethanol. The precipitates were then dried at room temperature for 24 h. The obtained nanocomposite was labelled as dextran-coated nCPs (2g DEX). A schematic diagram on the synthesis method is shown in Figure 2.

### 2.4. Experimental Procedure

The experiments on H_2_O_2_ release were carried out according to the following procedure: 100 mg of synthesized nCPs (0g DEX), nCPs (2g DEX) and nCPs coated with dextran (2g DEX) were dissolved in 100 mL of distilled water at room temperature with constant stirring to ensure good mixing of the solution. Then, 1.0 mL of the samples was taken at the specific time intervals and mixed with 5.0 mL of CeSO_4_, which was then allowed to react within 2 min. After this step, the mixture was analyzed using a UV spectrophotometer (DB-20, HALO) using the adsorption band at 480 nm. The H_2_O_2_ content of all synthesized materials was analyzed using the concentration of H_2_O_2_ release calculated using Equation (4):

(4) $$H_{2}O_{2}\;concentration=\frac{C_{0}V_{0}-C_{t}\left(V_{0}+V_{sample}\right)\times 0.017}{V_{sample}}\times 1000$$

where *C_o_* and *C_t_* indicate the concentration of CeSO_4_ at the beginning and at the given reaction time, *t,* respectively. *V_o_* represents the cerium sulfate standard solution volume (mL) and *V*_sample_ represents the sample volume (mL).

The synthesized nCPs (0g DEX), nCPs (2g DEX) and dextran-coated nCPs (2g DEX) were evaluated for degradation efficiencies by monitoring the degradation of doxycycline (DOX) in the aqueous phase. In typical degradation experiments, 60 mg of nCPs (0g DEX), nCPs (2g DEX) and dextran-coated nCPs (2g DEX) was added into 100 mL of an aqueous solution of DOX with an initial concentration of 60 mg/L. This reaction was followed by the addition of a pre-calculated dosage of FeSO_4_. Then, 3 mL of samples was taken at specific time intervals and the reaction was quickly stopped with Na_2_SO_3_. Then, the samples were centrifuged and the supernatant of the solution was analyzed using a UV spectrophotometer (DB-20, HALO) in 1cm quartz cuvettes at adsorption band 370 nm. Finally, the DOX degradation efficiencies were calculated from Equation (5):

(5) $$\mathrm{DOX}\;\mathrm{degradation}\;\mathrm{efficiency}\;\left(\%\right)=\frac{C_{0}-C_{t}}{C_{0}}\times 100$$

where *C*_0_ and *C_t_* represent the DOX concentrations at the initial time and DOX concentrations at the designated reaction time, *t*, respectively.

### 2.5. Analytical Method

The H_2_O_2_ release from nCPs (0g DEX), nCPs (2g DEX) and dextran-coated nCPs (2g DEX) was analyzed by measuring the H_2_O_2_ concentration at the desired time intervals using a UV spectrophotometer (DB-20, HALO). The wavelength used for this measurement was 480 nm.

The concentration of doxycycline and its degradation profile was determined by UV-visible spectroscopy (DB-20, HALO). The UV-visible absorption spectra of all samples were determined by full-scan analysis in the range of 250–500 nm (in 2 nm steps). The absorbance of doxycycline λ_max_ was determined at 370 nm and the spectra were saved for further investigations.

### 2.6. Characterization of nCPs (0g DEX), nCPs (2g DEX) and Dextran-Coated nCPs (2g DEX)

X-ray diffractometry (XRD) was used to identify the structure and phase analysis of the synthesized nCPs (0g DEX), nCPs (2g DEX) and dextran-coated nCPs (2g DEX) by using Shimadzu XRD-6000. The powders were scanned over the range of 2ϴ from 20° to 80° at a scanning speed of 4° min^−1^ using CuKα radiation (wavelength = 0.154 nm, 30 mA and 30 kV). The XRD spectra were analyzed with the software X′Pert HighScore Plus (PANalytical B.V., Almelo, Netherlands) and the full width at half maximum (FWHM) of the peaks was calculated. The surface morphology of the prepared nCPs (0g DEX), nCPs (2g DEX) and dextran-coated nCPs (2g DEX) was characterized by field emission scanning electron microscopy (FESEM) (NOVA NANOSEM 230, magnification: 500–300,000, Corvallis, OR, USA). The fourier transform infrared spectroscopy (FTIR) of the nCPs (0g DEX), nCPs (2g DEX) and dextran-coated nCPs (2g DEX) was analyzed by Bruker FTIR Spectrometer ALPHA II (wavelength range 4000–500 cm^−1^, (Bruker Corporation, Billerica, MA, USA) with a spectral resolution of 4 cm^−1^. Brunauer, Emmet and Teller (BET) analysis with nitrogen adsorption-desorption was used to characterize the textural properties of the synthesized nCPs (0g DEX), nCPs (2g DEX) and dextran-coated nCPs (2g DEX) with nitrogen adsorption-desorption isotherms degassed at 80 °C for 24 h in a nitrogen environment using the Autosorb-1 (Quantachrome Co., Hampshire, UK). Thermal stability tests were conducted in a temperature range of 25 °C to 800 °C using a Mettler Toledo (TGA/SDTA 851, Zurich, Switzerland) in a nitrogen atmosphere at a rate of 10 °C min^−1^. The particle size and polydispersity index (PDI) of the synthesized nCPs (0g DEX), nCPs (2g DEX) and dextran-coated nCPs (2g DEX) dispersed in ethanol were measured at a scattering angle of 90 using a nanoparticle sizer (Nano S, Malvern Instruments Ltd., Malvern, UK).

## 3. Results and Discussion

### 3.1. Characterization of nCPs (0g DEX) and nCPs (2g DEX)

**Figure 3:**
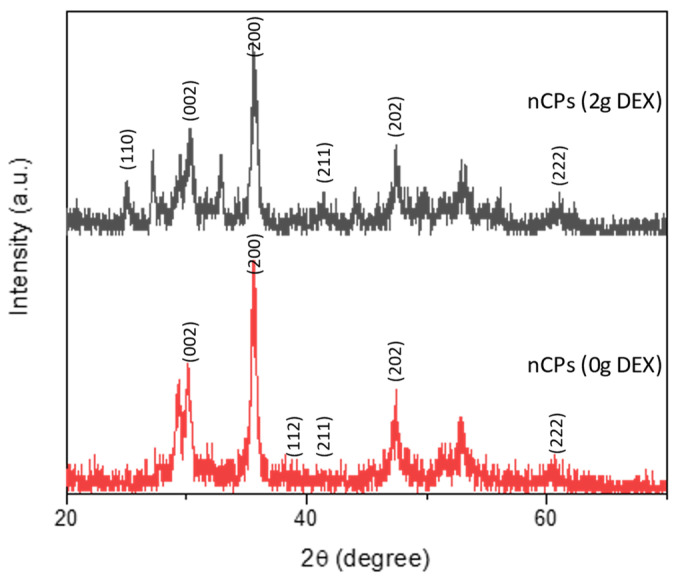
XRD of nCPs (0gDEX) and nCPs (2gDEX).

Figure 3 demonstrates the XRD patterns of the prepared nCPs (0g DEX) and nCPs (2g DEX). The dominant peaks in the prepared nCPs (0g DEX) were observed at 2θ = 30.33°, 35.67°, 39.55°, 43.03°, 47.41° and 60.83° and indexed as (0 0 2), (2 0 0), (1 1 2), (2 1 1), (2 0 2) and (2 2 2), respectively. The dominant peaks at 25.10°, 30.36°35.97°, 43.15°, 47.44° and 60.71° were observed in the nCPs (2g DEX) and indexed as (1 1 0), (0 0 2), (2 0 0), (2 1 1), (2 0 2) and (2 2 2) respectively. The tetragonal structure has been confirmed by both samples, which is in accordance with the standard set by the Joint Committee on Powder Diffraction Standards (JCPDS-03-0865) CaO_2_ and with the results from earlier studies. [35]. The average particle sizes of nCPs (0g DEX) and nCPs (2g DEX) were evaluated using the Debye–Scherer Equation (6):

(6) $$D=\frac{K\lambda }{\beta \cos\theta }$$

where *K* is the Scherrer constant (0.9), *D* is the mean particle size (nm), *λ* is the wavelength of the incident X-ray radiation (0.15406 nm), *θ* is the degree of the diffraction peak and *β* is the full width at half maximum (FWHM) of the XRD peak occurring at the diffraction angle (*θ*). The most intense common peak at (35°) with d-spacing (2.52A°) was used to determine the average particle sizes. The average particle size of nCPs (0g DEX) and nCPs (2g DEX) was 21.6 nm and 15.4 nm, respectively. As Figure 1 shows, the intensity peaks of the XRD pattern decrease with the addition of dextran. In addition, the peak of nCPs (2g DEX) also slightly broadens compared to the peaks of nCPs (0g DEX).

**Figure 4:**
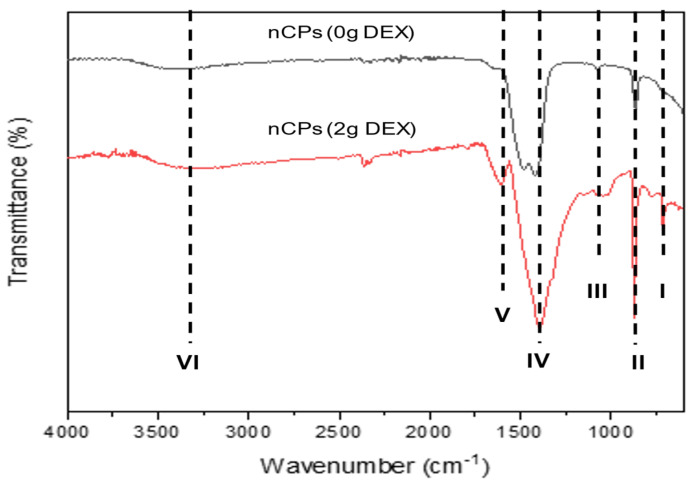
FTIR of nCPs (0gDEX) and nCPs (2gDEX).

The FTIR spectra of the nCPs (0g DEX) and the nCPs (2g DEX) synthesized by the co-precipitation method are shown in Figure 4. A small common absorption peak at 740–742 cm^−1^ marked at dashed line I and an intense peak marked at dashed line II at 850–870 cm^−1^ contributed to the O–O stretching vibrations [36]. The carbonate ion (CO_3_^2−^) was detected as a weak absorption peak between 1075 and 1080 cm^−1^, but this peak is relatively small (dashed line III). In the spectra for both nCPs (0g DEX) and nCPs (2g DEX), an intense peak near 1450–1470 cm^−1^ can be observed (dashed line IV). The peak indicated the presence of the bending vibration of O–Ca–O of CaO_2_ and the CO_3_^2−^ (carbonate) group of calcites. A signal at 1650 cm^−1^ at dashed line V was attributed to the stretching mode of the band of the H–O–H bending of residual free water in the nCPs (0g DEX) and nCPs (2g DEX), respectively. The bands at 3190–3490 cm^−1^ in dashed line VI correspond to the O–H mode of vibration. This was attributed to the vibrational mode O–H bond of the hydroxyl group that might come from water adsorbed by the nCPs (0g DEX) and nCPs (2g DEX) from the humid atmosphere or moisture from the sample itself.

**Figure 5:**
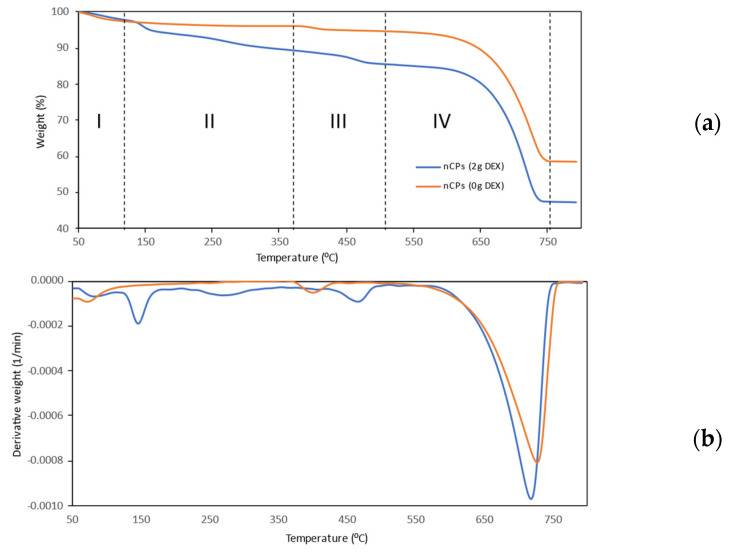
TGA (**a**) and DTG (**b**) curves of nCPs (0g DEX) and nCPs (2g DEX).

Figure 5a,b show the TGA thermogram analysis of nCPs (0g DEX) and nCPs (2g DEX) together with their DTG. The TG curves show that weight loss occurs in three stages for nCPs (0g DEX) and four stages for nCPs (2g DEX) in the temperature range of 50–800 °C. The initial stage of weight loss in the TG thermogram for nCPs (0g DEX) began between 50 and 100 °C and was caused by the dehydration of adsorbed water. This stage resulted in a loss of approximately 3.75% of the total weight. Due to the absence of dextran in the nCPs (0g DEX), stage II did not demonstrate any signs of weight reduction. Stage II showed no weight loss because no dextran was added to the nCPs (0g DEX). The second weight loss (stage III) showed a weight loss due to the decomposition of CaO2 starting at 375 °C and a weight loss of about 1.36%. The third weight loss (stage IV) was attributed to the decomposition of CaCO_3_ in the temperature range of 500–750 °C. In this stage, a weight loss of up to 36.23% was observed.

In the case of nCPs (2g DEX), the first stage of weight loss in the TGA curve represents the dehydration of water and 6.20% of weight loss between the temperatures of 50 and 140 °C. In addition, the second weight loss (stage II) coincided with the decomposition of the employed organic polymer, dextran. The TG curve indicated that dextran started to decompose at 150–350 °C [28]. Polysaccharide chain breakage (including dehydration, deamination, deacetylation, glycoside bond breaking and pyranose ring opening), evaporation and the removal of degradation products are all factors that contribute to the weight loss that occurs during this stage. In stage II, weight loss of up to 4.36% was observed. Next, the third weight loss (stage III) was observed at 370–500 °C, which was attributed to the decomposition of CaO_2_ with the liberation of oxygen. In stage III, weight loss of up to 4.27% was observed. The weight loss continued between 550–770 °C, which was attributed to the CaCO_3_ [24]. In stage IV, weight loss was observed up to 37.68%. The main factor for the formation of calcite is possibly due to the carbonation of Ca(OH)_2_, which occurs as a direct result of the hydrolysis of the precipitated calcium peroxide during synthesis.

Nitrogen adsorption-desorption measurements were used to characterize the structural properties of the prepared nCPs (0g DEX), nCPs (2g DEX) and dextran-coated nCPs (2g DEX). The surface area, pore size and pore volume of the prepared nCPs are listed in Table 1. The larger surface area (52.31 m^2^/g) of the nCPs (2g DEX) corresponded to a large pore size (65.13 nm) and pore volume (1.70 cm^3^/g) with a small average particle size compared to the nCPs (0g DEX), which have a slightly smaller surface area (41.13 m^2^/g), corresponding to a slightly smaller pore size (63.02 nm) and smaller pore volume (1.31 cm^3^/g). The small particle size of nCPs (2g DEX), which is 2.33 ± 0.81 nm, contributed to the high surface area compared to nCPs (0g DEX), which have average mean sizes of 4.19 ± 1.00 nm. In contrast, dextran-coated nCPs (2g DEX) had a small surface area due to the polymer coating on the nanoparticles, which resulted in an increase in average mean size of 154.70 ± 56.47 nm. As described in the FESEM analysis, this is likely due to the presence of clustered macrospheres. The high controlled-release efficiency of dextran-coated nCPs (2g DEX) due to the small surface area is consistent with the characterization of the dextran-coated nCPs (2g DEX) in the later sections. Moreover, it can be observed from Table 1 that dextran-coated nCPs (2g DEX) have the highest pore size and pore volume compared to nCPs (0g DEX) and nCPs (2g DEX). This could be due to the formation of an interconnected polymeric network with a high degree of porosity. These data suggest that the coating of dextran on nCPs (2g DEX) formed highly porous nanocomposites with a cross-linked network. In addition, nCPs (2g DEX) and nCPs (0g DEX) showed low controlled-release efficiency due to their high surface areas of 52.31 and 41.13 m^2^/g, respectively.

**Figure 6:**
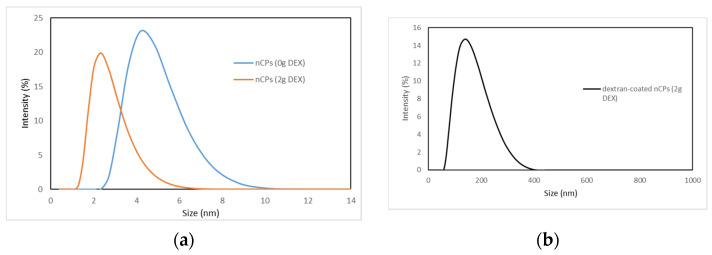
Particle size distributions of (**a**) nCPs (0g DEX) and nCPs (2g DEX) and (**b**) dextran-coated nCPs (2g DEX).

Dynamic light scattering (DLS) was used to measure the hydrodynamic sizes of nCPs (0g DEX), nCPs (2g DEX) and dextran-coated nCPs (2g DEX) dispersed in ethanol. The DLS analysis in Table 1 and Figure 6 shows narrow size distribution with an average hydrodynamic diameter of 4.19 ± 1.00 nm and 2.33 ± 0.81 nm for nCPs (0g DEX) and nCPs (2g DEX), respectively. In contrast to this, the observed average mean sizes of dextran-coated nCPs (2g DEX) (154.70 ± 56.47 nm) show the successful polymer coating of the particles with the dextran. As discussed in the FESEM section later, the increase in the mean size of dextran-coated nCPs (2g DEX) due to the multiple interactions of dextran on the surface of nCPs (2g DEX) facilitate their aggregation to form larger particles. As shown in Table 1, the PDI values for nCPs (0g DEX), nCPs (2g DEX) and dextran-coated nCPs (2g DEX) are 0.215, 0.398 and 0.203, respectively. The PDI values that are lower than 0.5 indicate that the nanoparticles are monodispersed. In addition, low PDI values also indicate that the nanoparticles have good aggregative stability. The lowest PDI value is shown by dextran-coated nCPs (2g DEX) and this is expected due to the utilization of dextran to stabilize and coat the nCPs during the synthesization process. In addition, the hydrodynamic sizes of nCPs (0g DEX) and nCPs (2g DEX) are in good agreement with their sizes calculated in XRD analysis.

**Figure 7:**
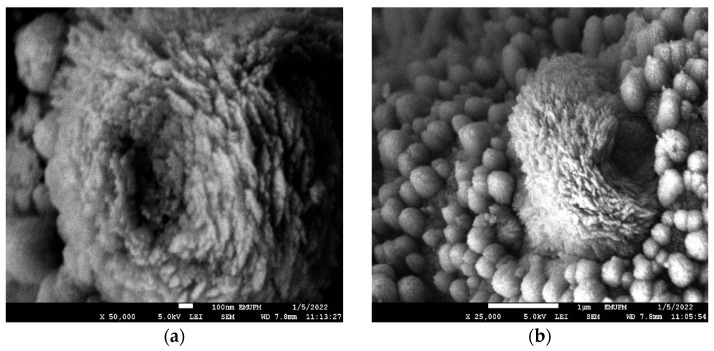

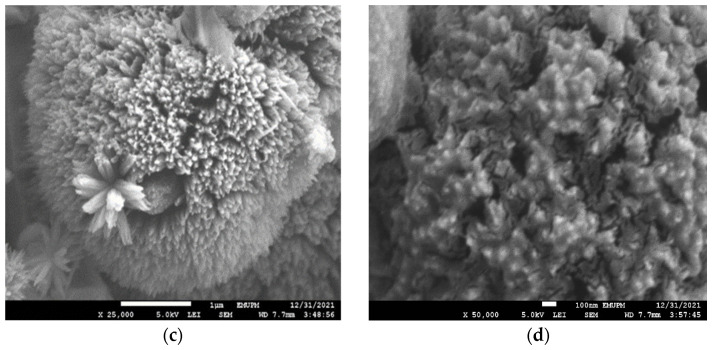
FESEM images of (**a**,**b**) nCPs (0g DEX) and (**c**,**d**) nCPs (2g DEX).

FESEM images were obtained to characterize the morphologies of nCPs (0g DEX) and nCPs (2g DEX). The results revealed an intriguing relationship between nCPs synthesized with and without the presence of dextran. The morphology and structure of nCPs (0g DEX) were shown in Figure 7a,b at different magnifications. From these images, it is shown that nCPs (0g DEX) prepared without any addition of dextran as a surface stabilizer were an irregular spherical shape with variation in size. In contrast, Figure 7c,d shows that the nCPs (2g DEX) synthesized by dextran were rod-like in shape and accumulated to form a flower-like shape. Moreover, from these images, it is clearly shown they were uniformly decorated. This result was in good agreement with Qi et al. (2013) who also obtained flower-like zinc oxide nanostructures when dextran was used as a stabilizer in the synthesis of nanoparticles [37]. In addition, it is evident that the presence of dextran in an adequate quantity is necessary for developing these unique flower-like nCPs. The nCPs were assisted in their transformation into nanorods by the presence of dextran. Since the nanorods have high surface energy due to the high surface/volume ratio, the rods tend to join together to create a relatively large petal to minimize the surface energy.

### 3.2. Characterization of Dextran-Coated nCPs (2g DEX)

TGA analysis, FTIR spectroscopy, BET, DLS and FESEM were used in this study to unequivocally detect the dextran coating on the surface of the dextran-coated nCPs (2g DEX).

**Figure 8:**
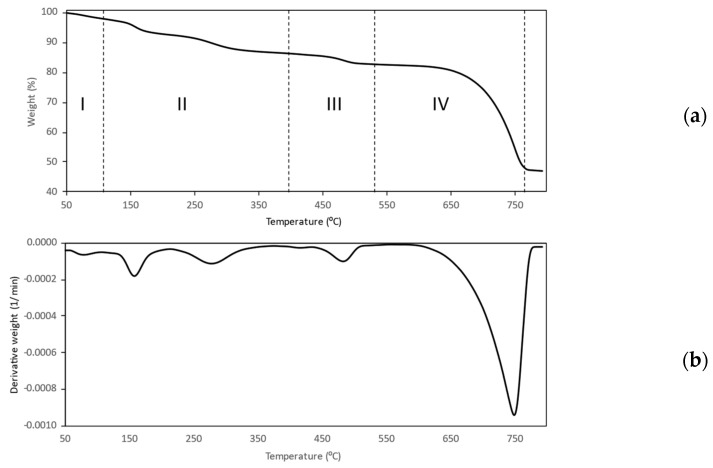
TG (**a**) and DTG (**b**) curves of dextran-coated nCPs (2g DEX).

The presence of a polymer coating on the dextran-coated nCPs (2g DEX) was confirmed by TGA analysis at temperatures between 50 to 800 °C. In order to facilitate a comparison with the TGA curve of nCPs (2g DEX) (previous section), the TGA curve was segmented into four stages, as shown in Figure 8a. The first weight loss (stage 1), which happened at 50–120 °C, was due to the loss of water, which has a weight loss of 7.16%. Stage II of the TGA curve for the dextran-coated nCPs (2g DEX) showed a weight loss of 6.24% due to the decomposition of dextran, which was approximately twice the weight loss (4.36 %) of the nCPs (2g DEX). This reveals that the dextran-coated nCPs (2g DEX) were successfully coated by the dextran. The coating of dextran on dextran-coated nCPs (2g DEX) was also confirmed by the large depth of the decomposition in stage II and stage III (more dextran content) and the short depth of the DTG peak (low CaO_2_ content), as shown in Figure 8b. In addition, based on the total weight of the samples used for TGA analysis, the percentage weight of the organic polymer in the coating of the nCPs (2g DEX) was calculated. The polymer weight of the dextran-coated nCPs (2g DEX) was 6.32%.

**Figure 9:**
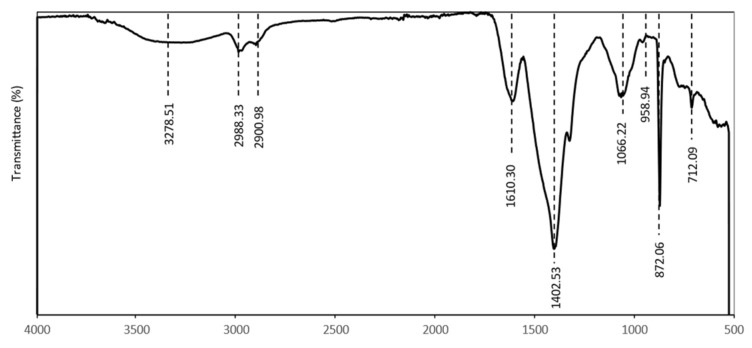
FTIR of dextran-coated nCPs (2g DEX).

Figure 9 shows the FTIR spectra of dextran-coated nCPs (2g DEX), which shows the effect of polymer coating on nCPs (2g DEX). The absorption peaks at 712 and 1066 cm^−1^ are characteristics of the structure of dextran, which confirmed the dextran coating on nCPs (2g DEX) [38]. All major signals at 872.06 and 1402.53 cm^−1^ remained as such in the spectra of nCPs (2g DEX). The fact that the coating was not affected by the persistent nature of nCPs is consistent with the results of previously published research [36,37]. Considering the FTIR spectroscopic results, the most likely mechanism for this coating is the hydrogen bonding of the dextran hydroxyl groups on the particle surface of the nCPs. The water lies between the dextran and the surface of the nCPs and forms inter- and intra-molecular hydrogen bonds. Other than that, the bands at 900–1000 cm^−1^ are related to the contribution of the C–O and C–C stretching vibrations. This confirms that the dextran-coated nCPs (2g DEX) contain dextran that consists of amylose and amylopectin, which are composed of a long chain of monosaccharide, a simple sugar derived from aldehyde, or ketone derivatives of straight-chain polyhydroxy alcohols with at least three carbon atoms. The absorption band at 2988.33 and 2900.98 cm^−1^ indicated the presence of methyl (–CH_3_) in the dextran structure. The absorption band from 3278.51 cm^−1^ was assigned to O–H vibrations.

**Figure 10:**
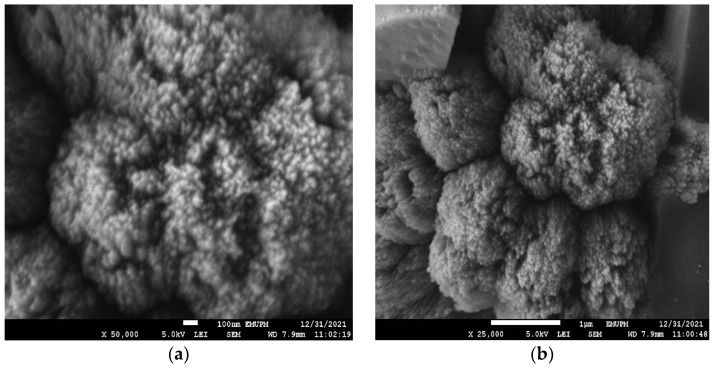
FESEM image of (**a**) and (**b**) dextran-coated nCPs (2g DEX) at different magnifications.

A distinctive behavior was exhibited by the dextran-coated nCPs (2g DEX), which had been synthesized by employing dextran as a surface stabilizer. As can be observed in Figure 10a,b, the nanoparticles aggregated into larger spheres when they came into contact with one another. The formation of the macrospheres was believed to be due to several interactions, including hydrogen bonding and the Van der Waals forces of the dextran. As a result, the surface area was relatively small, as discussed in the BET analysis, with average mean sizes of 154.7 ± 56.47 (Table 1).

### 3.3. H_2_O_2_ Controlled-Release Performance

**Figure 11:**
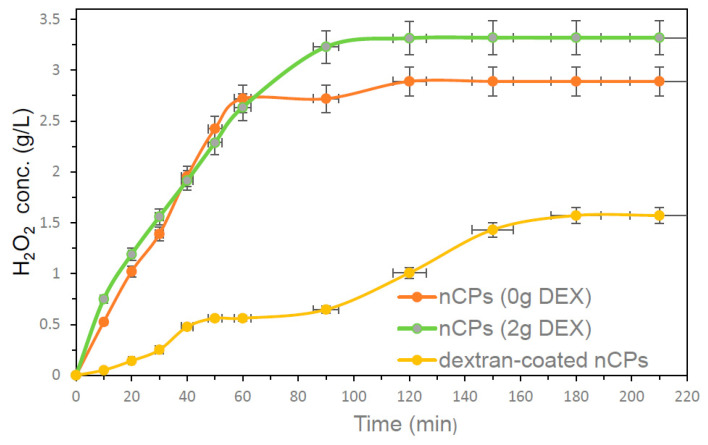
The H_2_O_2_ release profiles of nCPs and dextran-coated nCPs (DEX).

The release rates of H_2_O_2_ from the synthesized nCPs and dextran-coated nCPs (DEX) were measured and shown in Figure 11. The continuous release of H_2_O_2_ was observed for 210 min by dissolving 100 mg of nCPs and dextran-coated nCPs in 100 mL of distilled water without initial pH adjustment. The prepared nCPs showed fast H_2_O_2_ release behavior in the initial 10 min and kept increasing until up to 60 min. Both nCPs showed stable H_2_O_2_ release between 90 to 210 min. After 120 min, the highest H_2_O_2_ concentration released by nCPs (2g DEX) was 3.32 g/L, while it was only 2.89 g/L for nCPs (0g DEX). The maximum H_2_O_2_ concentration released by nCPs (2g DEX) was 3.32 g/L, while for nCPs (0g DEX) it was 2.89 g/L at 120 min, respectively. The released H_2_O_2_ release profile of dextran-coated nCPs (2g DEX) showed slow release up to 30 min, then increased and became stable at 180 min. Therefore, dextran-coated nCPs (2g DEX) showed excellent controlled-release performance with a maximum concentration of H_2_O_2_ of 1.57 g/L. The controlled-release efficiency for dextran-coated nCPs (2g DEX) can be analyzed using Equation (7) [24].

(7) $$\begin{array}{l} \mathrm{Controlled}\;\mathrm{release}\;\mathrm{efficiency}\;\mathrm{of}\;H_{2}O_{2}\;\left(\%\right)\\ =\frac{H_{2}O_{2}\;\mathrm{release}\;\left(\mathrm{nCPs}\right)-H_{2}O_{2}\;\mathrm{release}\;\left(\mathrm{dextran}-\mathrm{coated}\;\mathrm{nCPs}\right)}{H_{2}O_{2}\;\mathrm{release}\;\left(\mathrm{nCPs}\right)}\times 100 \end{array}$$

The controlled-release efficiency of H_2_O_2_ for dextran-coated nCPs (2g DEX) was 52.7%, which is quite good and in agreement with the result obtained by Ali et al. (2020) [24]. The evidence of good controlled-released efficiency was also confirmed by TGA and FTIR analyses. It is concluded from the H_2_O_2_ release profile that using dextran as an organic polymer to coat nCPs can attribute good controlled-release efficiency performance. This is consistent with the results of a number of other studies that have also shown that the synthesis of nCPs with polymer coating has the ability to control the release rates of H_2_O_2_ due to the effect of the coating [15,24,38].

### 3.4. DOX Degradation Performance

**Figure 12:**
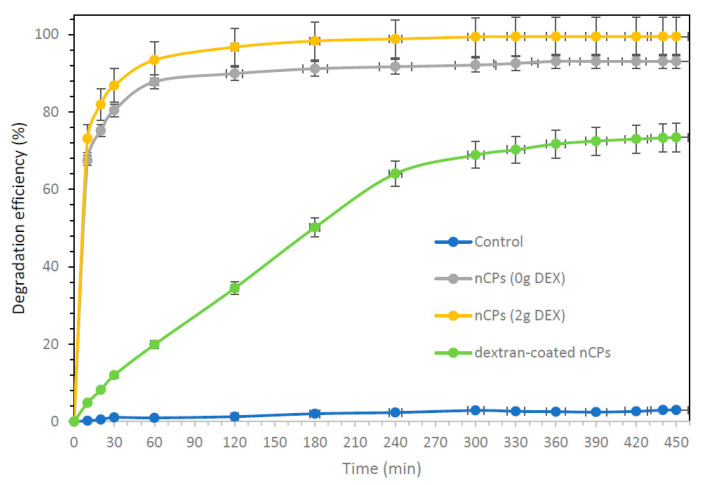
The degradation of DOX using nCPs (0g DEX), nCPs (2g DEX) and dextran-coated nCPs (2g DEX) catalyzed by Fe(II), concentration of DOX = 60 mg/L, concentration of Fe(II) = 1 mM, nCPs = 600 mg/L.

The performances of DOX degradation using synthesized nCPs (0g DEX), nCPs (2g DEX) and dextran-coated nCPs (2g DEX) are presented in Figure 12. The experiments were carried out at a constant initial DOX concentration of 60 mg/L at room temperature. As shown in Figure 12, over 99.5% and 93% of DOX were degraded by nCPs (2g DEX) and nCPs (0g DEX), respectively, after 450 min. It shows that nCPs (2g DEX) performed much better and effectively compared to the nCPs without any stabilizer. The better removal of DOX by nCPs (2g DEX) could mainly be due to the following reasons. Firstly, nCPs (2g DEX) could release up to 3.32 g/L of H2O2, while nCPs (0g DEX) only release up to 2.89 g/L of H_2_O_2_ at 210 min, respectively. Second, the oxidation ability of nCPs (2g DEX) is better due to their smaller average mean size (2.33 ± 0.81 nm) and slightly larger surface area (52.31 m^2^/g) compared to nCPs (0g DEX) with an average mean size of 4.19 ± 1.00 nm and surface area of 41.13 m^2^/g. Sun et al. (2019) also found that nCPs have better oxidation ability compared to conventional CaO_2_ due to the effect of the particle sizes [34].

The dextran-coated nCPs (2g DEX) show good controlled-release efficiency with slow DOX degradation. The slow DOX degradation was due to slow H_2_O_2_ release resulting from good controlled-release efficiency. It showed a DOX degradation of 73.4% after 450 min and a maximum of 78% after 24 h. The dextran-coated nCPs (2g DEX) showed DOX degradation efficiency of 12.00%, 34.50%, 64.06% and finally 73.40% after 30, 120, 240 and 450 min, respectively. The longevity of dextran-coated nCPs (2g DEX) made them a promising candidate for long-term efficacy in oxidation. This coated material was found to have high controlled-release efficiency and slow DOX degradation.

### 3.5. Possible Degradation Mechanisms

According to Equation (8), CaO_2_ will decompose into H_2_O_2_ when dissolved in water. The weak O–O bond of H_2_O_2_ will break down by Fe(II) Equation (9) to form hydroxyl radicals (•OH). Fe(II) acts as a catalyst and reacts rapidly with H_2_O_2_ to produce hydroxyl anion, Fe(III) and •OH with strong oxidizing properties. As shown in Equation (10), the hydroxyl radicals are responsible for the degradation of DOX to become intermediate products, carbon dioxide and water.

CaO_2_ + 2H_2_O → H_2_O_2_ + Ca(OH)_2_ (8)

H_2_O_2_ + Fe^2+^ → ●OH + OH^−^ + Fe^3+^ (9)

Doxycycline + ●OH → Intermediate products + CO_2_ + H_2_O (10)

**Figure 13:**
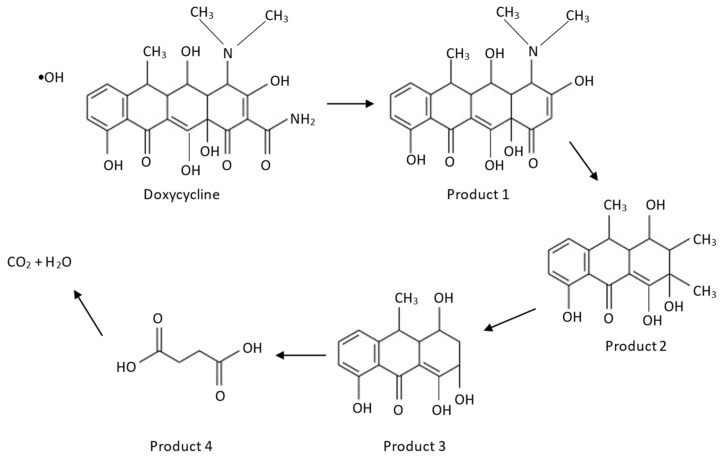
Proposed mechanism for the degradation of DOX by fenton reaction.

The possible mechanism for the degradation of DOX is illustrated in Figure 13. For the first degradation step, product 1 is formed through a deamidation reaction at the amide group in ring number four [39]. Next, product 2 is formed through the attack of the tertiary amine position on ring four via demethylation, followed by ring opening and an oxidative reaction [40]. For the third degradation step, product 3 is formed due to the bi-demethylation of the methyl groups. With further oxidation, product 4 is formed through ring opening and finally decomposes into CO_2_ and H_2_O [41].

## 4. Conclusions

The present research highlights the application of dextran as a surface stabilizer to prevent the irreversible agglomeration of synthesized nanoparticles as well as for the coating for controlled-release application. The synthesized nCPs (2g DEX) were characterized comprehensively using various techniques including X-ray diffraction (XRD), field emission scanning electron microscopy (FESEM), thermogravimetric analysis (TGA), Brunauer-Emmett-Teller (BET) analysis and dynamic light scattering (DLS). The synthesized nCPs (2g DEX) confirmed a smaller average mean size (2.33 ± 0.81nm) with high surface area (52.31 m^2^/g), slightly high pore size (65.13 nm) and pore volume (1.70 cm^3^/g) compared to the nCPs (0g DEX). Then, dextran-coated nCPs (2g DEX) were further synthesized by coating the synthesized nCPs (2g DEX) with dextran. The different characterization of dextran-coated nCPs (2g DEX) was been carried out, including field emission scanning electron microscopy (FESEM), thermogravimetric analysis (TGA), Brunauer–Emmett–Teller (BET) analysis and dynamic light scattering (DLS), to confirm polymer coating on dextran-coated nCPs (2g DEX). BET and DLS analysis demonstrated that dextran-coated nCPs (2g DEX) have a high average mean size (154.70 ± 56.47 nm), low surface area (23.96 m^2^/g), large pore volume (1.92 cm^3^/g) and large pore size (160.48 nm), supporting high polymer coating to the dextran-coated nCPs (2g DEX). Furthermore, dextran-coated nCPs (2g DEX) also show good aggregative stability as they are monodispersed and have the lowest PDI values (0.203) compared to nCPs (0g DEX) and nCPs (2g DEX). The controlled-release efficiency and DOX degradation efficiencies of the synthesized nCPs (0g DEX), nCPs (2g DEX) and dextran-coated nCPs (2g DEX) showed an inverse relationship. In summary, this study demonstrates that dextran can be applied as a surface stabilizer to reduce nanoparticle size as well as for the encapsulation of nanoparticles for controlled-release purposes. In addition, nCPs (2g DEX) are a feasible and potential option for the degradation and removal of DOX from wastewater, while dextran-coated nCPs (2g DEX) are proposed as a novel oxidant for long persistent applications in wastewater remediation applications. This work is potentially applicable for the oxidative decomposition of other contaminants such as non-steroidal anti-inflammatory drugs (NSAIDs) and organic pollutants in an aqueous environment, though much more work is needed to make these results applicable for wider ranges of data.

## Figures and Tables

**Table 1:** Physicochemical properties of the nCPs (0g DEX), nCPs (2g DEX) and dextran-coated nCPs (2g DEX).

Sample	Surface Area (m^2^/g)	Pore Size (nm)	Pore Volume (cm^3^/g)	Mean Size (nm)	PDI
nCPs (0g DEX)	41.13	63.02	1.31	4.19 ± 1.00	0.215
nCPs (2g DEX)	52.31	65.13	1.70	2.33 ± 0.81	0.398
Dextran-coated nCps (2g DEX)	23.96	160.48	1.92	154.70 ± 56.47	0.203

## Data Availability

The data presented in this study are available on request from the corresponding author.
